# Inteins as indicators of gene flow in the halobacteria

**DOI:** 10.3389/fmicb.2014.00299

**Published:** 2014-06-26

**Authors:** Shannon M. Soucy, Matthew S. Fullmer, R. Thane Papke, Johann Peter Gogarten

**Affiliations:** Department of Molecular and Cell Biology, University of ConnecticutStorrs, CT, USA

**Keywords:** gene symbiosis, genome as an ecosystem, inteins, mobile genetic elements, gene flow, horizontal gene transfer, halobacteria

## Abstract

This research uses inteins, a type of mobile genetic element, to infer patterns of gene transfer within the Halobacteria. We surveyed 118 genomes representing 26 genera of Halobacteria for intein sequences. We then used the presence-absence profile, sequence similarity and phylogenies from the inteins recovered to explore how intein distribution can provide insight on the dynamics of gene flow between closely related and divergent organisms. We identified 24 proteins in the Halobacteria that have been invaded by inteins at some point in their evolutionary history, including two proteins not previously reported to contain an intein. Furthermore, the size of an intein is used as a heuristic for the phase of the intein's life cycle. Larger size inteins are assumed to be the canonical two domain inteins, consisting of self-splicing and homing endonuclease domains (HEN); smaller sizes are assumed to have lost the HEN domain. For many halobacterial groups the consensus phylogenetic signal derived from intein sequences is compatible with vertical inheritance or with a strong gene transfer bias creating these clusters. Regardless, the coexistence of intein-free and intein-containing alleles reveal ongoing transfer and loss of inteins within these groups. Inteins were frequently shared with other Euryarchaeota and among the Bacteria, with members of the Cyanobacteria (*Cyanothece, Anabaena*), Bacteriodetes (*Salinibacter*), Betaproteobacteria (*Delftia, Acidovorax*), Firmicutes (*Halanaerobium*), Actinobacteria (*Longispora*), and Deinococcus-Thermus-group.

## Introduction

Inteins are self-splicing genetic parasites located in highly conserved sites of slowly evolving genes. They are found in all three domains of life and in viruses (Perler et al., 1997; Pietrokovski, 2001; Gogarten et al., 2002; Swithers et al., 2009). Similar to group I introns, inteins are often associated with a homing endonuclease (HEN). An important difference between inteins and introns is the timing of the splicing activity, which occurs immediately after transcription in introns and after translation in inteins (Hirata et al., 1990; Kane et al., 1990). The association with a HEN domain enables a cyclic invasion pattern, called the homing cycle (Goddard and Burt, 1999; Gogarten and Hilario, 2006). The homing cycle consists of three phases: intein invasion, intein fixation, and eventually loss of the intein enabling invasion to occur again. During invasion and fixation the intein splicing domains are associated with a HEN domain forming a canonical intein (hereafter referred to as a large intein); however, during the loss phase the function of the HEN is often disrupted and begins to degrade, generating a mini-intein. Simulations have shown that intein-containing and intein-free alleles can coexist in well mixed populations under some sets of parameters (Yahara et al., 2009; Barzel et al., 2011). Also, inteins with functioning HEN domains were inferred to have persisted in some eukaryotic lineages for several 100 million years (Butler et al., 2006; Gogarten and Hilario, 2006).

Inteins do not have an apparatus to penetrate the cell envelope. Therefore, they must rely on mechanisms in place within the population for insertion into the cell such as: conjugation, mating, generalized DNA uptake, and viruses or gene transfer agents (Lang et al., 2012). The faster-than-Mendelian inheritance of the large inteins (Gimble and Thorner, 1992), along with a nearly neutral fitness burden, enables these mobile elements to persist in organisms over evolutionary time as long as there are new populations to invade (Goddard and Burt, 1999; Gogarten and Hilario, 2006). Furthermore, the size of the intein (mini or large) provides information about the genomic mobility of the element as mini inteins are rarely integrated into the recipient's genome; whereas large inteins are more frequently integrated due to the activity of the HEN. The conservation of the recognition site provides an invasion target even in distantly related strains and species. Also, inteins have a higher substitution rate relative to their extein hosts (Swithers et al., 2013). This substitution rate gives rise to many evolutionarily informative sites when comparing a large collection of homologous inteins. In this work, we take advantage of these traits and survey the distribution of inteins in the Halobacteria, a highly recombinant class of halophilic Archaea (Williams et al., 2012) known to contain several intein alleles (Perler, 2002). We make use of 118 halobacterial genomes (Supplementary Table 1) and the previously reported and newly discovered intein alleles to survey networks of gene transfer within and outside the Halobacteria based on the presence-absence profile of the inteins, their sequence similarity, and the phylogenies reconstructed from intein sequences.

## Materials and methods

### Halobacterial intein sequence retrieval and alignment

Position specific scoring matrices (PSSMs) were created using the collection of all inteins from InBase, the Intein database and registry (Perler, 2002). A custom database was created with all inteins, and each intein was used as a seed to create a PSSM using the custom database. These PSSMs were then used as a seed for PSI-BLAST (Altschul, 1997) searches against each of the halobacterial genomes available from NCBI as of June 2013 as well as a private collection sequenced by our collaborators. To remove false positives, a size exclusion step was then performed on each protein sequence as an intein domain adds 100–700 aa to invaded protein sequences. Inteins were then aligned using Muscle (Edgar, 2004) with default parameters in the SeaView version 4.0 software package (Gouy et al., 2010). Insertions, which passed the size exclusion step, but did not contain splicing domains, were removed and the previous steps were repeated using the resulting dataset on a collection of private genomes from the Papke lab. Prottest 3.2 (Guindon et al., 2010; Darriba et al., 2011) was used to determine an appropriate substitution model for the intein sequences, the WAG model was favored and used for all subsequent trees for consistency. Once the collection of halobacterial inteins was complete, sequences were re-aligned using SATé (Liu et al., 2012) to generate a final alignment using MAFFT (Katoh and Standley, 2013) to align, Muscle (Edgar, 2004) to merge, RAXML(Stamatakis, 2014) for tree estimation, and a WAG model for each allele.

To determine the relationship among all halobacterial inteins, the inteins were aligned using Muscle (Edgar, 2004). Subsequently a tree was built using PhyML v3.0 (Guindon et al., 2010) using a WAG substitution model with a Gamma shape parameter and the proportion of invariant sites estimated from the data.

### Intein retrieval outside the halobacteria

Each halobacterial intein was used as a BLAST (Altschul et al., 1990) query against the non-redundant database on NCBI. Any match with an *e*-value better than 0.000001 was aligned to the dataset to which its query belonged. Sequences were then filtered based on the protein annotation and goodness of fit to the existing alignment. As an additional filtering step each match was used as a query against the non-redundant database and the majority BLAST hit annotations were used to verify the protein identity, as annotations are not always reliable. Remaining sequences were aligned using Clustal Omega 1.1.0 (Sievers et al., 2011) with the profile alignment option in SeaView 4.0 (Gouy et al., 2010). Maximum-likelihood trees were built using PhyML (Guindon et al., 2010) with the WAG model, and rates estimated from the data.

To assess the relative contribution of different genera represented in each intein allele sequence data set, a stacked column graph was created. Sequence density was calculated for each intein allele by dividing the number of intein sequences in each genus by the number of total intein sequences in that allele.

### Symbiotic state assignment

Intein sequence length was used to determine symbiotic state. For each intein allele the length of the intein sequence was determined. A cutoff length for mini-intein assignment was based on the presence of a gap in intein lengths greater than 100 amino acids within an allele. The third intein state “no-intein” was assigned where the intein was clearly absent from the orthologous protein containing an intein in any of the halobacterial genomes examined. Additionally, once an intein was noted as a mini-intein the alignment was analyzed to ensure the gaps in these sequences correspond to the location of the HEN domain.

### Ribosomal protein reference tree

Alignments of 55 ribosomal protein for 21 Halobacteria (Williams et al., 2012) were used to find orthologous proteins in the genomes used in this work. In-house python scripts (data file 1) were used to concatenate the alignments, and PhyML v3.0 (Guindon et al., 2010) was used to build a tree. The tree used the WAG substitution model with the Gamma shape parameter and the proportion of invariant sites and base frequencies estimated from the data.

### Bayesian clustering with intein sequences

A concatenation of an intein presence-absence matrix and alignments for each intein allele were generated using in-house python scripts (data file 1). MrBayes version 3.2.1 (Ronquist et al., 2012) was then used to perform a clustering analysis using a partition allowing for character states in the presence-absence matrix and sequence information for each intein allele. The prior for the character portion of the data matrix used a symmetrical Dirichlet distribution with an exponential (1.0), and variable rates so each column was considered independent of the others. The likelihood for the character portion of the alignment used variable coding and 5 beta categories. The prior for the protein sequences in the alignment used a fixed WAG substitution model, with state frequencies estimated from the data, and the likelihood settings used a Gamma shape parameter and the proportion of invariant sites estimated from the data.

## Results

### Halobacterial inteins

**Figure 1:**
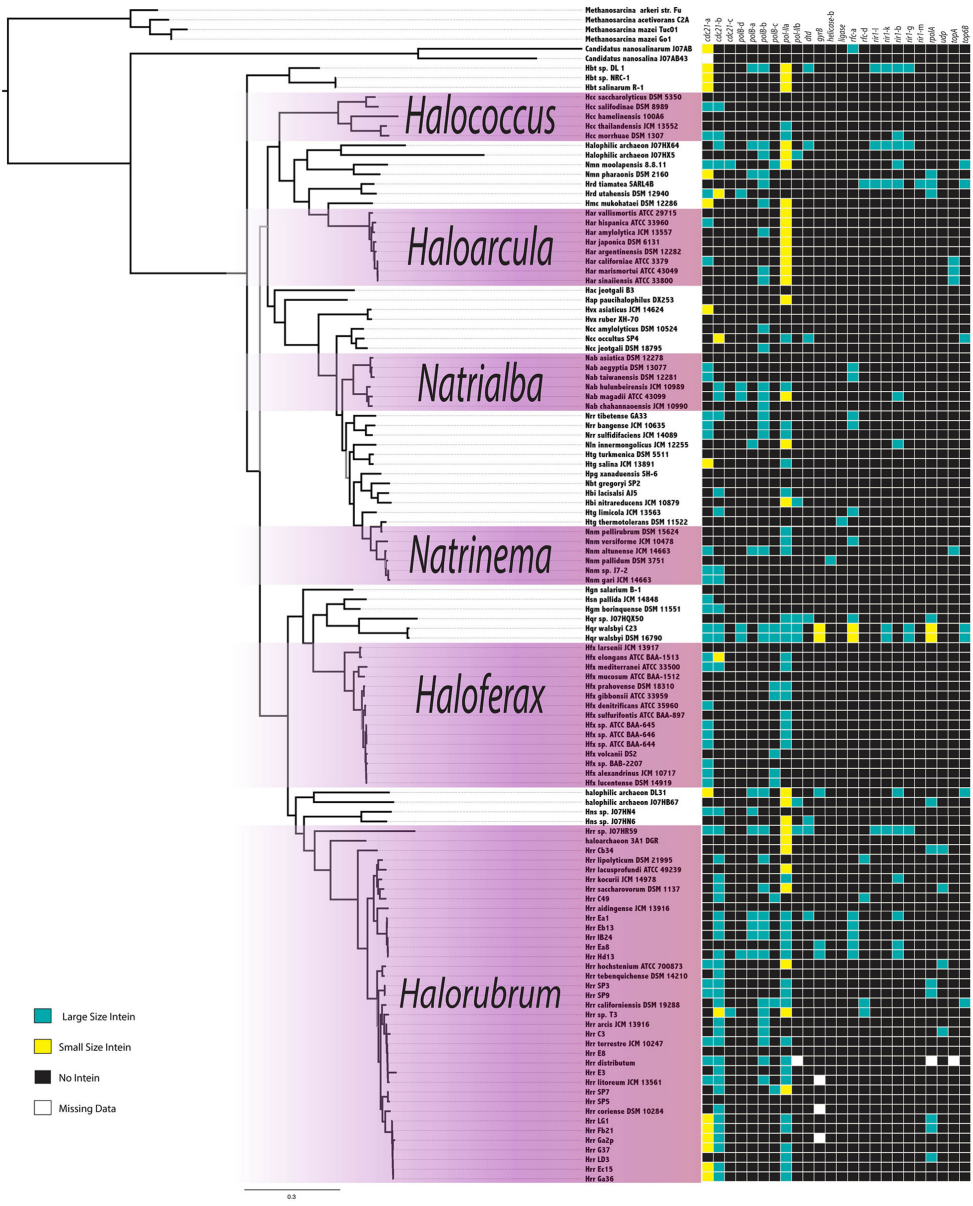
**Intein Invasion Pattern in the Halobacteria**. Intein pattern of presence-absence is mapped onto the tips of a ribosomal reference tree, teal boxes indicate the presence of a full size intein, yellow boxes indicate the presence of a mini-intein, black boxes indicate the absence of an intein, and white boxes indicate missing data. Purple shaded boxes indicate the genera with more than five species represented on the tree. Nodes with bootstrap support <70 are in gray.

The intein content of a collection of halobacterial genomes was analyzed using an intein-allele-specific PSSM. This survey revealed 13 genes in the Halobacteria invaded by inteins at 24 distinct positions (intein alleles) (Table 1). Seven of these intein alleles were not previously reported in the Halobacteria, and two of the seven have not previously been reported to harbor inteins: a DNA ligase gene involved in double strand break repair, and a deaminase gene involved in nucleotide metabolism (Table 1). To determine if vertical inheritance was accountable for the distribution of intein alleles, the presence–absence matrix of intein alleles was mapped onto a reference phylogeny (Figure 1). Clearly, intein presence-absence is not concordant with the ribosomal protein phylogeny, implicating abundant horizontal genetic transfer (HGT) in creating the observed distribution. The presence of multiple intein alleles in the majority of genomes (70%) might be interpreted to suggest that inteins could spread locally within a single genome.

**Table 1:** **Exteins in the halobacteria**.

**Intein allele**	**Extein annotation**
*cdc21*-a	Cell division control protein 21
*cdc21*-b	
*cdc21*-c	
*polB*-d	DNA polymerase B1
*polB*-a	
*polB*-b	
*polB*-c	
*pol-II*a	DNA polymerase II large subunit
*pol-II*b^*^	
dtd^**^	Deoxycytadine triphosphate deaminase
*gyrB*	DNA gyrase subunit B
*helicase*-b^*^	ATP-dependent helicase
ligase^**^	ATP-dependent DNA ligase I
*rfc*-a	Replication factor C small subunit
*rfc*-d^*^	
*rir1*-l^*^	Ribonucleoside-diphosphate reductase
*rir1*-k	
*rir1*-b	
*rir1*-g	
*rir1*-m^*^	
*rpolA*	DNA-directed RNA polymerase subunit A
*udp*	UDP-glucose 6-dehydrogenase
*topA*	DNA topoisomerase I
*top6B*	DNA topoisomerase VI subunit B

* *Denotes intein alleles discovered in this work*.

** *Denotes extein sequences not previously reported to be invaded by an intein*.

### Intein propagation within the halobacteria

**Figure 2:**
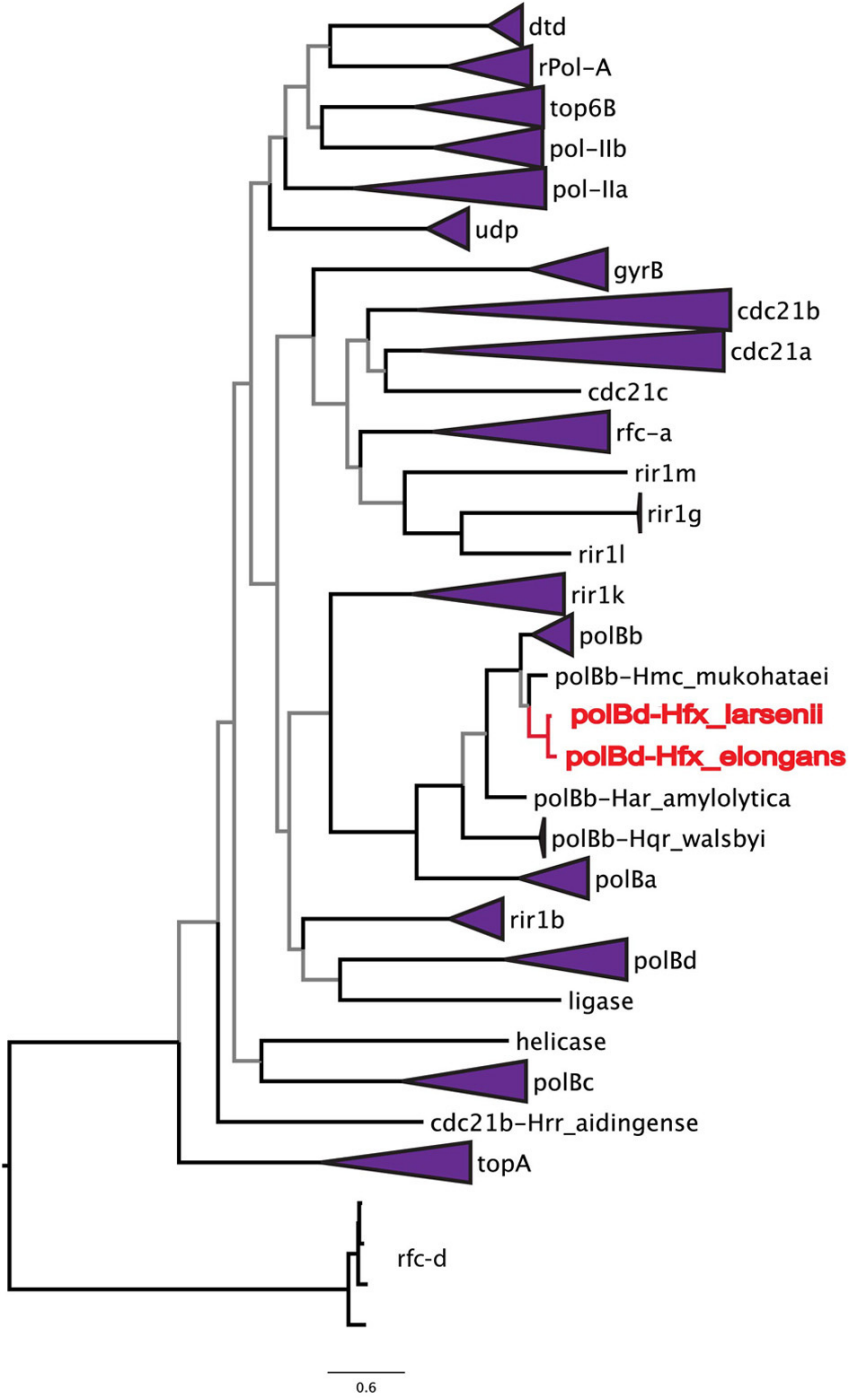
**Relationships among intein alleles in the Halobacteria**. This tree depicts the phylogenetic relationships among intein alleles in the Halobacteria. Inteins that clustered in concordance with the allele were collapsed to a single node, and labeled with the name of the intein allele. Two *polB*-d sequences did not group with the *polB*-d allele, and instead are located amongst the *polB*-c alleles these are indicated in red. Nodes with bootstrap support <70 are colored gray.

To address the possibility of inteins moving locally within a genome, the phylogenetic relationships among all halobacterial intein sequences were analyzed (Figure 2). All of the intein alleles form highly supported clusters with others of the same type, with the exception of two sequences: the *polB*-c inteins of *Haloferax larsenii* and *Haloferax elongans* group inside the *polB*-b intein allele cluster; however, this node is poorly supported (59/100 bootstraps) indicating this relationship could be an artifact produced by poor resolution of the relationships that connect various intein alleles. Furthermore, there is poor support linking all of the intein allele clusters together (less than 70% bootstrap support), indicating sequence conversion (an intein invading an ectopic or atypical locus) between intein alleles, even within the same host protein, is uncommon. Among the inteins analyzed here, at most one invasion of an ectopic site is supported by the data, confirming that this type of event is rare (Perler et al., 1997; Gogarten et al., 2002). These data indicate that HGT is the only plausible explanation for the large number of different intein alleles in this class of organisms. Incongruence between the presence of inteins and ribosomal phylogeny also support this conclusion.

### Bayesian phylogenetic analysis of inteins

**Figure 3:**
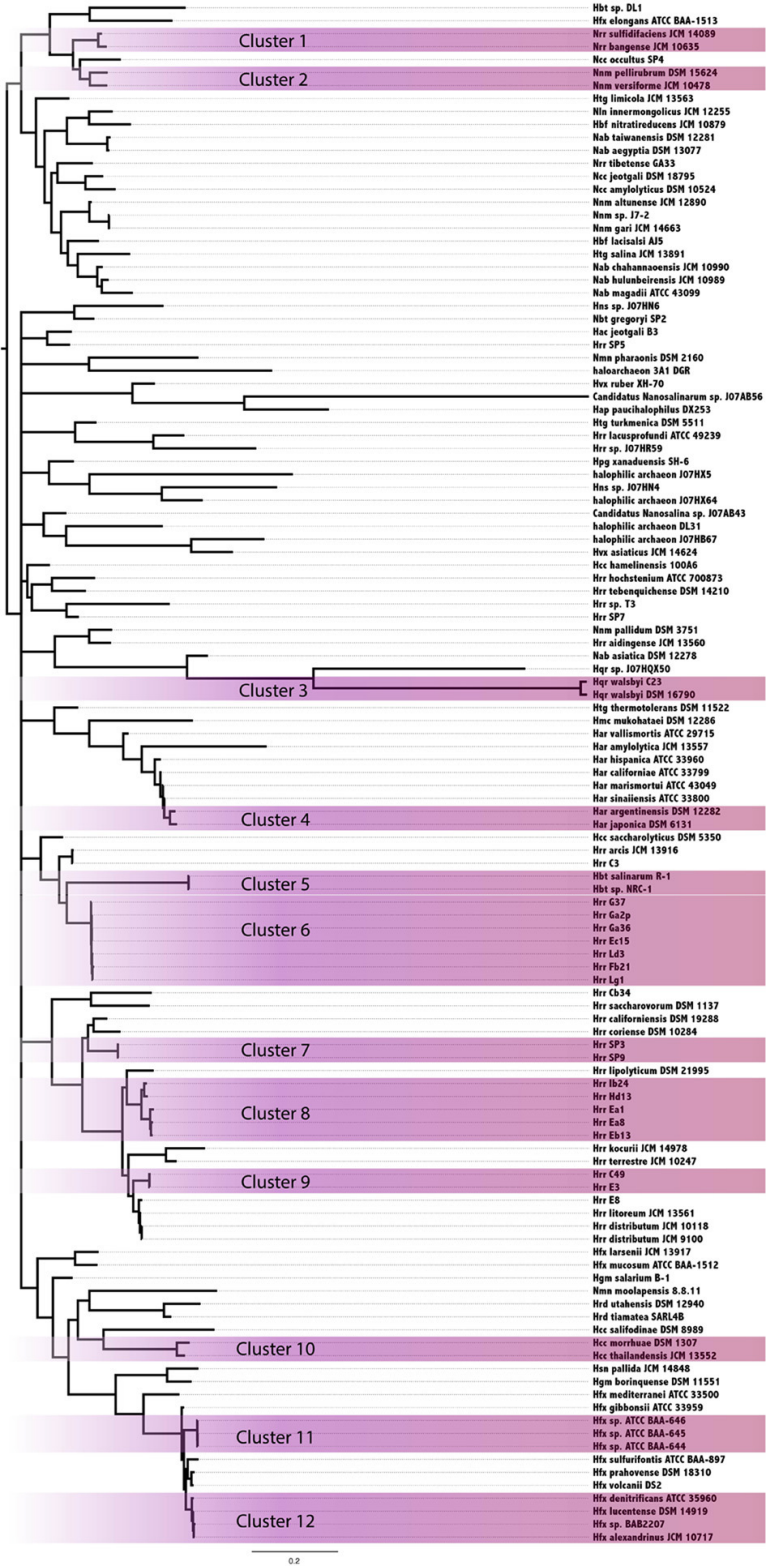
**Clustering of Halobacteria based on intein sequences and distribution**. Halobacteria were clustered based on intein sequences and the distribution in each genome. Clusters with posterior probability >95% are shaded purple.

In an attempt to resolve the local events (transfers and vertical inheritance within the Halobacteria) that gave rise to the observed intein distribution in the Halobacteria, a Bayesian analysis based on the intein sequences for each allele and on the presence-absence pattern was performed (Figure 3). In this analysis two organisms may group together because they both inherited inteins from a common ancestor, or because an intein was recently transferred between them. The paucity of well-supported nodes (nodes with 0.95 or greater posterior probability were considered well-supported) in part reflects the extent to which our sample is biased toward very similar sequences (31% of halobacterial genomes in this study are from *Halorubrum*). Most of the well-supported clusters in the Bayesian tree also occur in the reference tree, suggesting these inteins may be the result of shared vertical inheritance. However, many of these clusters do not have identical intein profiles (clusters 1, 6, 8, and 10), thus HGT between close relatives is a better explanation than vertical inheritance for these clusters. Only three of the clusters, 2, 9, and 12, have branching orders that are different from those observed in the reference tree indicating HGT. Cluster 2 is made up of *Natrinema* spp. *pellirubrum* and *versiforme* which share only the *pol-II*a intein. In the reference tree *Nnm. versiforme* groups with the rest of the *Natrinema*, and *Nnm. pellirubrum* groups with *Haloterrigena thermotolerans*. *Natrinema sp*. J7-2 is the only other member of the *Natrinema* that has an intein in the *pol-II*a position, but the intein in this species is 14 aa shorter than the intein shared by *Nnm. pellirubrum* and *Nnm. versiforme*. *Htg. thermotolerans* shares no inteins with *Nnm. pellirubrum*. Cluster 9 is made up of *Halorubrum* spp. C49 and E3, which share only the *cdc21*-b intein. In the reference tree *Hrr*. E3 groups with *Halorubrum litoreum* and the two share the *pol-II*a intein allele, but no others. *Hrr*. C49 groups with *Halorubrum saccharovorum* and they do not share any inteins. Cluster 12 is made up of *Haloferax* spp. *denitrificans, lucentense, alexandrinus*, and *Haloferax sp*. BAB2207, which all have an intein in the *cdc21*-a position. In the reference tree *Hfx. lucentense, Hfx*. sp. BAB2207, and *Hfx. alexandrinus* all group together, but *Hfx. denitrificans* groups with *Haloferax sulfurifontis*, and they do not share any inteins. The lack of shared inteins between clusters in the reference tree and differences among the inteins shared in these clusters cause these divergences in this tree as compared to the reference tree. This may indicate that the taxa in the Bayesian clusters are exchange partners, or that they share unsampled intermediate exchange partners. Additionally, the majority of clusters share 2 or fewer intein alleles between all members of the cluster (eight out of 12 clusters). The two clusters that share the most intein alleles between all members are Cluster 3, made up of *Haloqudratum walsbyi* strains DSM 16790 and C23 with 13 shared intein alleles, and cluster 7 made up of *Halorubrum* spp. strains SP3 and SP9 sharing 4 intein alleles. Both of these clusters have branching patterns identical to those on the reference tree, indicating that phylogenetic proximity plays a significant role in intein distribution.

Members of the *Halorubrum* genus, not surprisingly, were highly represented in the clusters (four of 12 total). All four of the clusters show a geographic bias. Clusters 6, 8, and 9 were all isolated from the Aran-Bidgol lake in Iran, and cluster 7 was isolated from the Sedom Ponds in Israel (Atanasova et al., 2012). Branch lengths in all of these clusters are very small, suggesting these populations are well mixed with respect to intein sequences. Geography does not seem to play a strong role in linking other well-supported clusters based on intein sequences. Furthermore, evidence of clustering based on geography in the *Halorubrum* is less interesting than the clear separation between groups isolated from the same location (cluster 6, 8, and 9). This separation of species of *Halorubrum* from the same location is echoed in the reference tree, and taken together with the short branch lengths in these clusters indicate that population structure plays a strong role in gene sharing at least for this location (see Fullmer et al., 2014 for in depth discussion). Increased geographical sampling could reveal similar trends in other locations.

### Intein homing in the halobacteria

The existence of a singleton in an intein allele in the genomes analyzed could represent intein invasion from outside the Halobacteria; but could also be due to incomplete sampling. To investigate the phylogenetic distance of invasion events responsible for the observed distribution of inteins, the halobacterial inteins were used as queries to search for homologous sequences in the non-redundant database (Altschul et al., 1990). Intein sequences that matched the alleles in the Halobacteria were found in other Euryarchaeota (but not Crenarchaeota), and Bacteria (Table 2). To ascertain whether homing occurred between the Halobacteria and organisms outside the Halobacteria, a maximum likelihood tree was built for each intein allele. The tree topologies were evaluated with respect to the halobacterial inteins. If the halobacterial inteins in the tree were monophyletic it was assumed that except for the initial invasion gene flow for that intein allele occurred within the Halobacteria exclusively. If the halobacterial inteins were polyphyletic, invasion events that generated the observed distribution likely involved organisms outside the Halobacteria either as donors or as recipients. The majority of intein trees, 83%, were monophyletic, reinforcing the idea that recombination is more successful between closely related organisms (Gogarten et al., 2002; Zhaxybayeva et al., 2006; Andam et al., 2010; Papke and Gogarten, 2012; Williams et al., 2012). Interestingly, for trees where the Halobacteria were polyphyletic, the organisms interrupting the clade were Bacteria for two out of the four polyphyletic intein alleles. The sample size restricts building strong claims about HGT between the Halobacteria and the Bacteria. However, this claim is supported by previous evidence of gene exchange between the Bacteria and the Halobacteria (Ng et al., 2000; Khomyakova et al., 2011).

**Table 2:** **Taxonomic distribution in each intein allele**.

**Intein allele**	**Tree topology**	**Halobacteria**	**Bacteria**	**Other Euryarchaeota**
*cdc21-a*	Monophyletic	55	4	16
*cdc21-c*	Monophyletic	1	0	0
dtd^**^	Monophyletic	6	0	0
*gyrB*	Monophyletic	6	19	1
helicase-b^*^	Monophyletic	1	2	1
ligase^**^	Monophyletic	1	0	0
pol-IIb^*^	Monophyletic	9	0	1
*polB-d*	Monophyletic	6	0	1
*rfc-a*	Monophyletic	16	0	13
rfc-d^*^	Monophyletic	5	0	0
*rir1-b*	Monophyletic	15	55	5
*rir1-g*	Monophyletic	4	15	0
*rir1-k*	Monophyletic	5	1	0
rir1-l^*^	Monophyletic	3	3	0
*rpolA*	Monophyletic	10	0	0
*top6B*	Monophyletic	8	0	0
*topA*	Monophyletic	4	0	1
*udp*	Monophyletic	7	2	6
rir1-m^*^	Monophyletic	1	4	0
*polB-c*	Monophyletic	20	1	1
*polB-a*	Polyphyletic-bacteria	16	2	1
*polB-b*	Polyphyletic-bacteria	38	3	0
*pol-II*a	Polyphyletic-Euryarchaeota	75	0	16
*cdc21-b*	Polyphyletic-Euryarchaeota	51	1	3

* *Denotes intein alleles discovered in this work*.

** *Denotes exteins discovered in this work*.

**Figure 4:**
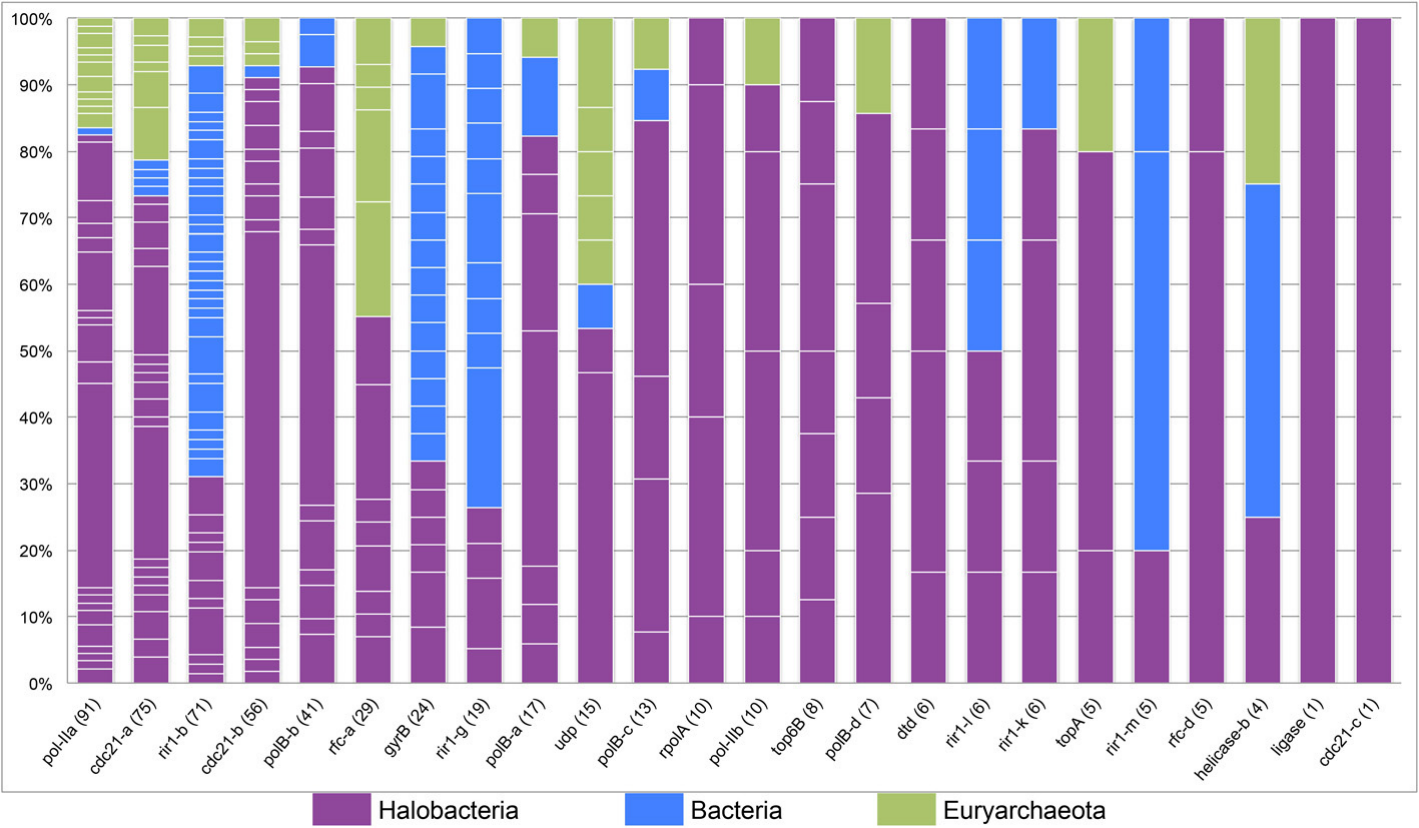
**Phylogenetic diversity in halobacterial intein alleles**. A stacked column graph depicts the representation of the Halobacteria (in purple), the Bacteria (in blue), and other Euryarchaeota (in green). Intein alleles are ordered by the number of intein sequences recovered for each allele, which is reported in parenthesis after the intein allele name on the x-axis. The number of genera for each intein allele is indicated by the number of breaks in the column (white lines) and the height of each of the fragments that make up a column indicate the proportion of sequences in that allele found in a particular genus.

The tight clustering of halobacterial intein sequences and short branches between closely related strains indicate that in the majority cases inteins are inherited vertically or are transferred between closely related strains, and that successful invasion across large genetic distances is rare. Thus, intein alleles that are found in many different genera have been active for many generations, enabling invasion of many lineages, and accumulating examples of rare invasion events such as those that cross domain boundaries. Conversely, a lack of taxonomic diversity cannot be interpreted as a recent invasion as sampling limitations could be responsible for the paucity of samples in that intein allele. While many factors influence the success of intein transfer between divergent organisms, phylogenetic diversity of the organisms invaded by a particular intein allele also is a reflection of the time the intein allele has been present in a linage. Furthermore, a high density of intein sequences in a particular domain or group of genera can be used to determine the most likely reservoir for the circulating intein allele. A stacked column chart was used to quantify the representation of each of the genera in each of the intein alleles (Figure 4). Five intein alleles, *cdc21*b, *pol-II*a, *polB*b, *cdc21*a, and *rfc*-d, show polarity in intein density favoring the Halobacteria (specifically *Halorubrum*) as the reservoir for the intein population. This is not surprising as the data indicate that the majority of intein transfer in the Halobacteria is within the class. Additionally, the diversity in five of the intein alleles, *helicase*-b, *cdc21*a, *gyrB*, *rir*1-b, and *udp*, suggests these intein populations may be more ancient than the others in this study as they have had time to accumulate rare, long distance transfers such that the diversity within them spans both class and domain boundaries. Interestingly, the *helicase*-b intein was only recently discovered in this study, though the diversity in the allele gives the impression that this intein has been around for a long time.

### Transfer of inteins between halobacterial and non-halobacterial lineages

**Figure 5:**
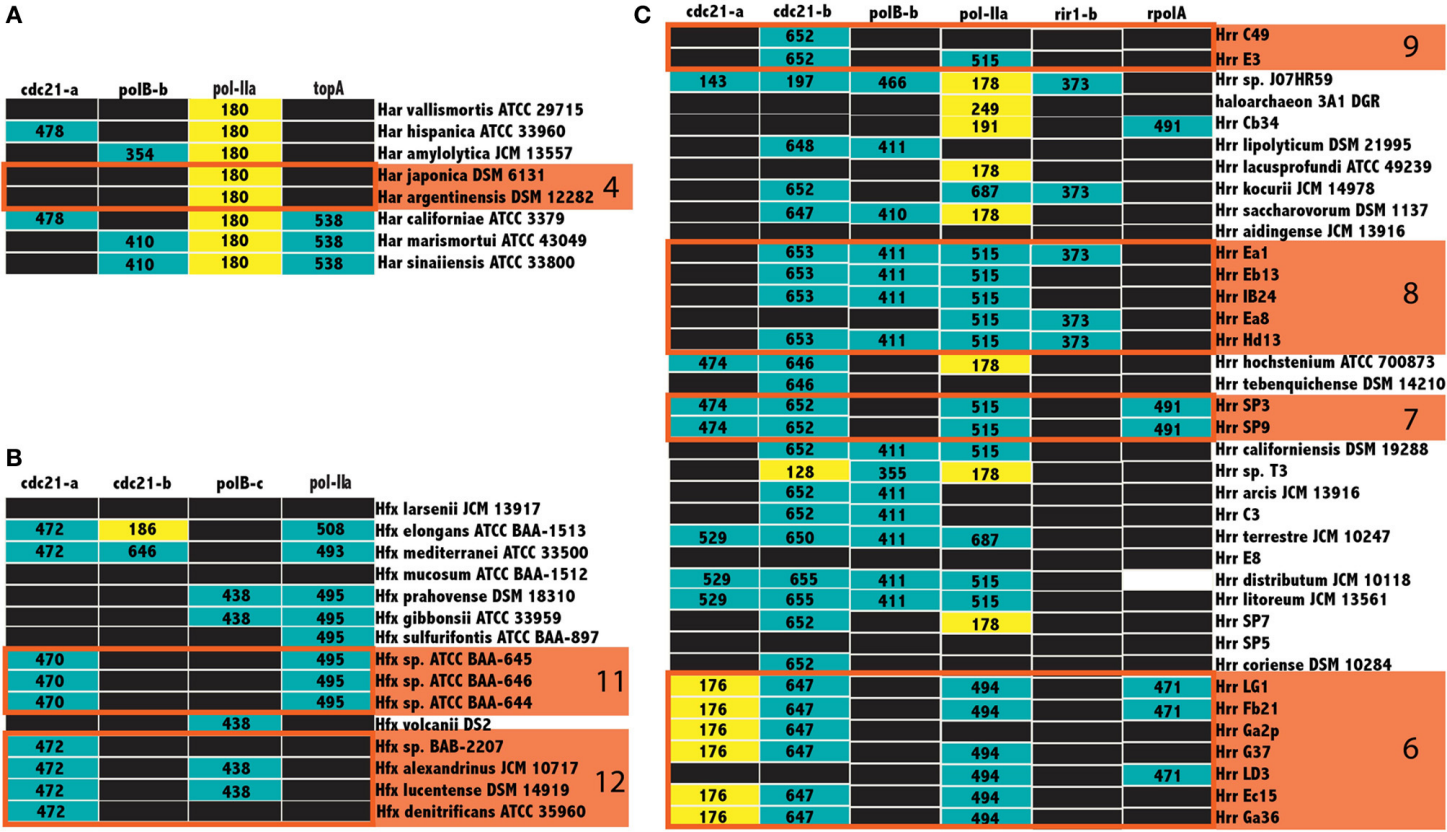
**Intein size distributions in the *Haloferax, Haloarcula*, and *Halorubrum***. The size of inteins in the *Haloarcula* **(A)**, *Haloferax* **(B)**, and *Halorubrum* **(C)** are indicated in the column corresponding to the intein allele. Mini-inteins are colored yellow, large inteins are colored teal, black boxes indicate no intein, and white boxes indicate missing data, clusters from Figure 3 are indicated by numbered orange boxes. The *cdc21-*a, and b sequences for *Halorubrum* sp. J07HR59, though smaller than the rest, cannot be considered mini-inteins, as the intein sequences in these positions are not complete.

Not all inteins are transferred equally; the efficiency of intein invasion is affected largely by the state of the intein. The HEN domain in canonical inteins is required to induce a double strand break and the subsequent homologous repair that results in invasion (Pietrokovski, 2001). Thus, mini-inteins that have lost a functioning HEN domain are mainly transferred vertically (they may be transferred horizontally together with the host gene). If an intein containing allele has been fixed in a population, either a precise deletion of the mini intein encoding DNA could remove the intein from the population or homologous replacement by an intein-free allele transferred from outside the population. Thus, mini-inteins are maintained through strong purifying selection, because any mutation that decreases the self-splicing activity decreases the availability of the host protein (Barzel et al., 2011). The intein states were determined to infer patterns of homing in the Halobacteria. The size of inteins in each allele, along with the position of gaps in the alignment relative to the HEN domain were used as a heuristic for assigning mini-intein status. In most cases there was a clear separation in the distribution of intein lengths (at least 100 amino acids difference in length). The size of more populated intein alleles within the three genera of the Halobacteria with the largest number of available genomes, *Haloarcula*, *Haloferax*, and *Halorubrum*, were recorded in a matrix of intein alleles (Figure 5). Many intein alleles show a considerable size variation. This variability can be attributed to the accumulation of insertions and deletions in various lineages over time, which in some lineages leads to loss of the HEN domain. Notably, there is no variability in the size of intein sequences shared by the clusters recovered in the Bayesian analysis (orange boxes Figure 5) reinforcing the claim of ongoing gene exchange in these clusters.

Invasion from outside the Halobacteria is one explanation for the polyphyletic topology observed in some halobacterial intein alleles. To determine when these homing events could have occurred, the state of each intein was determined and mapped onto polyphyletic intein allele trees: the results of that analysis are summarized in Table 3, with mini-inteins indicated with a star (^*^), and inteins that group within the Halobacteria indicated by a tilde (~) next to the name of the organism. Many of the intein sequences (5 out of 11) from taxa outside the Halobacteria that interrupt the clade are large-inteins, indicating that interactions between these taxa and the Halobacteria, though rare are ongoing (Table 3). Though the assignment of direction of transfers is extremely preliminary as limited sampling can affect the assignment of direction of transfer, there are some cases with an overwhelming signal where the majority of sequences originate from the Halobacteria, or the Bacteria in the case of *rir1*-m. The mixture of mini and large inteins represented in all of the intein alleles imply most of these inteins are active in the Halobacteria, and notably involve a wide distribution of taxonomic exchange partners.

**Table 3:** **Protein sequence identifiers for intein sequences**.

**Intein allele**	**Species name**	**Accession number**	**Phylum**
*cdc21*-a	*Archaeoglobus profundus* DSM 5631	YP_004340760.1	Euryarchaeota
	^*^Archaeoglobus veneficus SNP6	YP_003400528.1	Euryarchaeota
	^*^Candidatus Methanomassiliicoccus intestinalis Issoire Mx1	YP_008072558.1	Euryarchaeota
	^*^Croccosphaera watsonii	WP_021836378.1	Cyanobacteria
	^*^Ferroglobus placidus DSM 10642	YP_003435419.1	Euryarchaeota
	^*^Halarchaeum acidiphilum	WP_020220725.1	Halobacteria
	^*^Lamprocystis purpurea	WP_020504136.1	Gammaproteobacteria
	^*^Methanomassiliicoccus luminyensis	WP_019178416.1	Euryarchaeota
	^*^Methanothermococcus okinawensis IH1	YP_004576471.1	Euryarchaeota
	*Nocardia asteroides* NBRC 15531	GAD83132.1	Actinobacteria
	*Nocardiopsis potens*	WP_020380316.1	Actinobacteria
	*Pyrococcus abyssi* GE5	NP_127115.1	Euryarchaeota
	^*^Pyrococcus furiosus DSM 3638	NP_578211.1	Euryarchaeota
	^*^Pyrococcus horikoshii OT3	NP_142122.1	Euryarchaeota
	^*^Pyrococcus sp. NA2	YP_004424138.1	Euryarchaeota
	*Thermococcus litoralis* DSM 5473	YP_008429717.1	Euryarchaeota
	^*^Thermococcus onnurineus NA1	YP_002306424.1	Euryarchaeota
	^*^Thermococcus sibiricus MM 739	YP_002994932.1	Euryarchaeota
	^*^Thermococcus sp. AM4	YP_002582218.1	Euryarchaeota
	^*^Thermococcus sp. CL1	YP_006424652.1	Euryarchaeota
	^*^Thermococcus zilligii	WP_010479121.1	Euryarchaeota
	*Halorubrum* sp. SP3	KJ_865687.1	Halobacteria
	*Halorubrum* sp. SP9	KJ_865689.1	Halobacteria
*cdc21*-b	^*^Cyanothece sp. PCC 7822	YP_003887897.1	Cyanobacteria
	*Halarchaeum acidiphilum*	WP_020220725.1	Halobacteria
	^*^Candidatus Methanomassiliicoccus intestinalis Issoire-Mx1	YP_008072558.1	Euryarchaeota
	^*^Methanomassiliicoccus luminyensis	WP_019178416.1	Euryarchaeota
	^~^*Thermococcus barophilus*	YP_004070279.1	Euryarchaeota
	*Halorubrum* sp. SP3	KJ_865687.1	Halobacteria
	*Halorubrum* sp. SP7	KJ_865688.1	Halobacteria
	*Halorubrum* sp. SP9	KJ_865689.1	Halobacteria
*polB*-d	*Archaeoglobus profundus* DSM 5631	YP_003400528.1	Euryarchaeota
*polB*-a	^~^*Salinibacter ruber* M8	YP_003572085.1	Bacteroidetes
	^~^*Salinibacter ruber* DSM 13885	YP_446104.1	Bacteroidetes
	^~^*Halarchaeum acidiphilum*	WP_020678478.1	Halobacteria
	^~^*Methanoculleus bourgensis*	YP_006544623.1	Euryarchaeota
*polB*-b	*Halosimplex carlsbadense*	WP_006885382.1	Halobacteria
	^*^^~^*Salinibacter ruber* M8	YP_003572085.1	Bacteroidetes
	^*^^~^*Salinibacter ruber* DSM 13885	YP_446104.1	Bacteroidetes
	^*^^~^*Halanaerobium saccharolyticum*	WP_005489097.1	Firmicutes
	*Halarchaeum acidiphilum*	WP_020678478.1	Halobacteria
*polB*-c	^*^^~^*Thermus scotoductus*	YP_004202875.1	Deinococcus-Thermus
	^*^^~^*Methanotorris igneus* Kol 5	YP_004483799.1	Euryarchaeota
	*Halorubrum* sp. SP7	KJ_865686.1	Halobacteria
*pol-II*a	*Archaeoglobus veneficus* SNP6	YP_004341738.1	Euryarchaeota
	*Halosimplex carlsbadense*	WP_006882195.1	Halobacteria
	^*^Methanocaldococcus infernus ME	YP_003616947.1	Euryarchaeota
	*Methanococcus aeolicus*	ABU41683.1	Euryarchaeota
	^*^Methanoculleus bourgensis MS2	YP_006544019.1	Euryarchaeota
	^*^Methanoculleus marisnigri JR-1	YP_001048029.1	Euryarchaeota
	*Methanofollis liminatans*	WP_004037227.1	Euryarchaeota
	^*^Methanolinea tarda	WP_007314808.1	Euryarchaeota
	^*^Methanoplanus limicola	WP_004076782.1	Euryarchaeota
	^*^Methanoplanus petrolearius DSM 11571	YP_003893638.1	Euryarchaeota
	*Methanoregula boonei* 6A8	YP_001403293.1	Euryarchaeota
	^~^*Methanoregula fomicica* SMSP	YP_007242862.1	Euryarchaeota
	*Methanosphaerula palustris* E1-9c	YP_002467270.1	Euryarchaeota
	^*^Metahnospirillum hungatei JF-1	YP_503855.1	Euryarchaeota
	^*^Pyrococcus horikoshii OT3	NP_142130.1	Euryarchaeota
	^*^Thermococcus gammatolerans EJ3	YP_002958492.1	Euryarchaeota
	^*^Thermococcus sibiricus MM 739	YP_002994988.1	Euryarchaeota
	uncultured haloarchaeon	ABQ75865.1	Halobacteria
	*Halorubrum* sp. SP3	KJ_865692.1	Halobacteria
	*Halorubrum* sp. SP7	KJ_865690.1	Halobacteria
	*Halorubrum* sp. SP9	KJ_564691.1	Halobacteria
*pol-IIb*	*Halosimplex carlsbadense*	WP_006882195.1	Halobacteria
	^*^Pyrococcus abyssi GE5	YP_004624494.1	Euryarchaeota
	uncultured haloarchaeon	ABQ75865.1	Halobacteria
*gyrB*	*Allochromatium vinosum* DSM 180	YP_003443943.1	Gammaproteobacteria
	*Anabaena* sp. 90	YP_006997726	Cyanobacteria
	^*^Anabaena sp. PCC 7108	WP_016950132.1	Cyanobacteria
	*Bacillus subtilis* BEST7613	BAM51471.1	Firmicutes
	*Calothrix* sp. PCC 7103	WP_019489451.1	Cyanobacteria
	*Coleofasciculus chthonoplastes*	WP_006099284.1	Cyanobacteria
	^*^Cylindrospermopsis reciborskii	WP_006276716.1	Cyanobacteria
	^*^Dactylococcopsis slaina PCC 8305	YP_007173052.1	Cyanobacteria
	*Halarchaeum acidiphilum*	WP_021780646.1	Halobacteria
	*Methanomassiliicoccus luminyensis*	WP_019178436.1	Euryarchaeota
	*Microcystis aeruginosa*	WP_002774451.1	Cyanobacteria
	*Moorea producens*	WP_008190351.1	Cyanobacteria
	*Oscillatoria* sp. PCC 10802	WP_017715151.1	Cyanobacteria
	*Pleurocapsa* sp. PCC 7319	WP_019509077.1	Cyanobacteria
	*Prochlorothrix hollandica*	WP_017710941.1	Cyanobacteria
	*Raphidiopsis brookii*	WP_009342634.1	Cyanobacteria
	*Rivularia* sp. PCC 7116	YP_007054134.1	Cyanobacteria
	*Saccharothrix espanaensis* DSM 44229	YP_007037469.1	Actinobacteria
	*Synechocystis* sp. PCC 6803	NP_441040.1	Cyanobacteria
	*Trichodesium erythraeum* IMS101	YP_723459.1	Cyanobacteria
	uncultured bacterium	EKD46222.1	
*helicase*-b	^*^Bacillus amyloliqufaciens TA208	YP_005540906.1	Firmicutes
	^*^Bacillus subtilis	WP_017696872.1	Firmicutes
	Nanoarchaeota archaeon SCGC AAA011-L22	WP_018204386.1	
*rfc*-a	*Methanocaldococcus jannaschii* DSM 2661	NP_248426.1	Euryarchaeota
	*Methanocaldococcus* sp. FS406	YP_003458055.1	Euryarchaeota
	*Methanothermococcus okinawensis* IH1	YP_004576337.1	Euryarchaeota
	^*^Methanotorris formicicus	WP_007044297.1	Euryarchaeota
	^*^Pyrococcus abyssi GE5	NP_125803.1	Euryarchaeota
	^*^Pyrococcus furiosus DSM 3638	NP_577822.1	Euryarchaeota
	^*^Pyrococcus horikoshii OT3	NP_142122.1	Euryarchaeota
	^*^Pyrococcus sp. ST04	YP_006353924.1	Euryarchaeota
	^*^Thermococcus kodakorensis KOD1	YP_184631.1	Euryarchaeota
	^*^Thermococcus litoralis DSM 5473	YP_008428897.1	Euryarchaeota
	*Thermococcus* sp. 4557	YP_004763272.1	Euryarchaeota
	*Thermococcus* sp. AM4	YP_002582171.1	Euryarchaeota
	^*^Thermococcus sp. CL1	YP_006425306.1	Euryarchaeota
*rpol*A	*Halorubrum* sp. SP3	KJ_865684.1	Halobacteria
	*Halorubrum* sp. SP9	KJ_865685.1	Halobacteria
*rir1*-l	*Chloroherpeton thalassium* ATCC 35110	YP_001995975.1	Chlorobi
	*Tepidanaerobacter acetatoxydans* Re1	YP_007273179.1	Firmicutes
	uncultured Chloroflexi bacterium	BAL53207.1	Chloroflexi
*rir1*-k	*Deinococcus peraridilitoris* DSM 19664	YP_007181218.1	Deinococcus-Thermus
*rir1*-b	*Acidovorax avenae* subsp. avenae ATCC 19860	YP_004233126.1	Betaproteobacteria
	*Acidovorax* sp. CF316	WP_007856012.1	Betaproteobacteria
	*Acidovorax* sp. NO-1	WP_008903130.1	Betaproteobacteria
	*Actinomadura atramentaria*	WP_019631066.1	Actinobacteria
	*Alicyclobacillus pohliae*	WP_018131875.1	Firmicutes
	*Aminomonas paucivorans*	WP_006300529.1	Synergistetes
	*Ammonifex degensii* KC4	WP_006300529.1	Firmicutes
	*Arhodomonas aquaeolei*	WP_018718131.1	Gammaproteobacteria
	*Bacillus licheniformis*	WP_016885361.1	Firmicutes
	*Bacillus subtilis*	WP_017697104.1	Firmicutes
	*Calothrix* sp. PCC 6303	YP_007136749.1	Cyanobacteria
	*Candidatus* Chloracidobacterium thermophilum B	YP_004863563.1	Acidobacteria
	*Candidatus* Desulforudis audaxviator MP104C	YP_001717412.1	Firmicutes
	*Clostridiaceae bacterium* L21-TH-D2	WP_006314960.1	Firmicutes
	*Deinococcus radiodurans* R1	NP_296095.1	Deinococcus-Thermus
	*Delftia acidovorans*	WP_016451949.1	Betaproteobacteria
	*Delftia* sp. Cs1-4	YP_004490724.1	Betaproteobacteria
	*Desulfitobacterium hafniense*	WP_005810476.1	Firmicutes
	*Desulfovibrio magneticus* RS-1	YP_002955841.1	Deltaproteobacteria
	*Desulfovibrio* sp. U5L	WP_009106508.1	Deltaproteobacteria
	*Ferroplasma acidarmanus* fer1	YP_008141532.1	Euryarchaeota
	*Ferroplasma* sp. Type II	WP_021787573.1	Euryarchaeota
	*Halomonas anticariensis*	WP_016418429.1	Gammaproteobacteria
	*Halomonas jeotgali*	WP_017429019.1	Gammaproteobacteria
	*Halomonas smyrnensis*	WP_016854101.1	Gammaproteobacteria
	*Mahella australiensis* 50-1 BON	YP_004462974.1	Firmicutes
	*Marinobacter lipolyticus*	WP_018405479.1	Gammaproteobacteria
	*Methanofollis liminatans*	WP_004040239.1	Euryarchaeota
	*Methylobacter marinus*	WP_020160338.1	Gammaproteobacteria
	*Methylococcus capsulatus*	WP_017366201.1	Gammaproteobacteria
	*Methylomicrobium buryatense*	WP_017841702.1	Gammaproteobacteria
	nanoarchaeote Nst1	WP_004578017.1	
	*Nocardiopsis halotolerans*	WP_017572347.1	Actinobacteria
	*Polaromonas* sp. JS666	CAJ57177.1	Cyanobacteria
	*Pseudanabaena* sp. PCC 6802	WP_019499030.1	Cyanobacteria
	*Pseudanabaena* sp. PCC 7367	YP_007101092.1	Cyanobacteria
	*Rhodanobacter fulvus*	WP_007082010.1	Gammaproteobacteria
	*Rhodanobacter* sp. 2APBS1	YP_007588821.1	Gammaproteobacteria
	*Rhodanobacter thiooxydans*	WP_008437232.1	Gammaproteobacteria
	*Rhodothermus marinus* SG0.5JP17-172	YP_004824118.1	Bacteroidetes
	*Staphylococcus aureus*	WP_016187732.1	Firmicutes
	*Synechococcus elongatus* PCC 6301	CAJ57178.1	Cyanobacteria
	*Synechococcus elongatus* PCC 7942	YP_400626.1	Cyanobacteria
	*Synechococcus* sp. PCC 6312	YP_007060778.1	Cyanobacteria
	*Thermoanaerobacterium saccharolyticum* JW/SL-YS485	YP_006391581.1	Firmicutes
	*Thermoanaerobacterium thermosaccharolyticum* DSM 571	YP_003851043.1	Firmicutes
	*Thermobrachium celere*	WP_018663796.1	Firmicutes
	*Thermococcus kodakarensis* KOD1	YP_184312.1	Euryarchaeota
	*Thermodesulfatator indicus* DSM 15286	YP_004625205.1	Thermodesulfobacteria
	*Thermovirga lienii* DSM 17291	YP_004932130.1	Deinococcus-Thermus
	*Thermus igniterrae*	WP_018110436.1	Deinococcus-Thermus
	*Thermus thermophilus* HB8	CAJ57170.1	Deinococcus-Thermus
	*Thioalkalivibrio* sp. ALE11	WP_019570879.1	Gammaproteobacteria
	*Thioalkalivibrio* sp. ALE30	WP_018881426.1	Gammaproteobacteria
	*Thioalkalivibrio* sp. HL-Eb18	WP_017926201.1	Gammaproteobacteria
	*Thioalkalivibrio* sp. K90mix	YP_003459507.1	Gammaproteobacteria
	uncultured bacterium	EKE25755.1	
	*Xanthomonas* sp. SHU199	WP_017907463.1	Gammaproteobacteria
	*Xanthomonas* sp. SHU308	WP_017915139.1	Gammaproteobacteria
	zeta proteobacterium SCGC AB-604-B04	WP_018280466.1	Zetaproteobacteria
*rir1-g*	*Chloroherpeton thalassium* ATCC 35110	YP_001995975.1	Chlorobi
	*Deinococcus aquatillis*	WP_019011777.1	Deinococcus-Thermus
	*Halothece* sp. PCC 7418	YP_007166732.1	Cyanobacteria
	*Klebsiella pneumoniae*	WP_021313783.1	Gammaproteobacteria
	*Nocardiopsis dassonvillei* subsp. Dassonvillei DSM 43111	YP_003681238.1	Actinobacteria
	*Nocardiopsis* sp. CNS639	WP_019609645.1	Actinobacteria
	*Rhodothermus marinus* SG0.5JP17-172	YP_004826277.1	Bacteroidetes
	*Tepidanaerobacter acetatoxydans* Re1	YP_007273179.1	Firmicutes
	*Thermomonospora curvata* DSM 43183	YP_003299200.1	Actinobacteria
	*Thermus thermophilus* HB27	YP_005899.1	Deinococcus-Thermus
	*Thermus thermophilus* HB8	CAJ57173.1	Deinococcus-Thermus
	*Thermus thermophilus* JL-18	YP_006059430.1	Deinococcus-Thermus
	*Thermus thermophilus* SG0.5JP17-16	YP_005639869.1	Deinococcus-Thermus
	*Trichodesium erythraeum* IMS101	YP_720358.1	Cyanobacteria
	uncultured Chloroflexi bacterium	BAL53207.1	Chloroflexi
*rir1*-m	*Thermus aquaticus*	WP_003044118.1	Deinococcus-Thermus
	*Thermus thermophilus* HB-8	CAJ57173.1	Deinococcus-Thermus
	*Thermus thermophilus* SG0.5JP17-16	YP_005639869.1	Deinococcus-Thermus
	uncultured Chloroflexi bacterium	BAL53207.1	Chloroflexi
*udp*	*Fervidibacteria bacterium* JGI 0000001-G10	WP_020250137.1	
	*Dictyglomus thermophilum* H-6-12	YP_002250310.1	Dictyglomi
	*Methanocaldococcus jannaschii* DSM 2661	NP_248048.1	Euryarchaeota
	*Methanocaldococcus vulcanis* M7	YP_003246412.1	Euryarchaeota
	*Methanococcus aeolicus* Nankai-3	YP_001324612.1	Euryarchaeota
	*Methanothermococcus okinawensis* IH1	YP_004575831.1	Euryarchaeota
	*Methanotorris igneus* Kol 5	WP_007044255.1	Euryarchaeota
	*Thermococus gammatolerans* EJ3	YP_002960518.1	Euryarchaeota
*topA*	*Methanotorris igneus* Kol 5	WP_007044255.1	Euryarchaeota
*top6B*	*Halarchaeum acidiphilum*	WP_021780130.1	Halobacterium

* *Indicates the intein detected is a mini-intein*.

~ *Indicates taxa that grouped within the halobacterial intein sequences*.

## Discussion

The importance of HGT throughout the tree of life demands the development of a system to monitor gene-flow within and between populations. This research provides fundamental evidence that mobile elements such as inteins can be used to uncover gene flow networks. Inteins have a unique combination of traits that make them ideal tools to study evolution in microbial populations. They have a naturally wide phylogenetic distribution, enabling detection of HGT between distantly related taxa. This is demonstrated in this work by the intein trees where the Halobacteria were polyphyletic (*pol-II*a, *polB*-a, *polB*-b, and *cdc21*b) indicating intein transfer between the Halobacteria and the taxa that interrupt them, as well as by data from other studies where intein transfer has been detected across phyla and domains (Butler et al., 2006; Swithers et al., 2013). Inteins also have a high substitution rate relative to their extein hosts, and a propensity for accumulating insertions and deletions, which makes detection of transfers between close relatives (generally a difficult task) possible; for example, transfer within the *Halorubrum* clusters shown in Figure 3. Inteins can be associated with a HEN domain. If they are, they possess the ability to invade intein-free alleles following transfer; if they are not, they rely mainly on vertical inheritance together with the host gene, and the occasional transfer of the host gene. One intein allele, *pol-II*a, is widely distributed in the Halobacteria and there are many examples of mini-intein sequences in this allele. These data suggest that invasion of this allele occurred early in the evolution of the Halobacteria, and that the intein may have been lost in some lineages, but retained as a mini intein in most of the genomes surveyed here. This could also be true for the *cdc21*-a intein; however, the distribution is not as diverse, and considerably fewer mini-inteins were detected. This is more suggestive of an intein that has been active in the Halobacteria for a long period of time, with the different intein states (empty target site, target site invaded by an intein with active HEN, target site occupied by an intein without functioning HEN; Yahara et al., 2009; Barzel et al., 2011) existing and co-existing in different halobacterial lineages.

The genomes analyzed in this work were cultured from salty water and soil samples around the world. The diverse background of the genomes may contribute to the spotty distribution of intein alleles (Figure 1). However, genomes isolated from the same location show variation as well (Figure 3) (Fullmer et al., 2014), reinforcing the notion that inteins are currently actively propagating in and being eliminated from halobacterial populations. Additionally, previous data have shown recombination occurs at a higher rate than mutation within the Halobacteria, and very little linkage between genes is detected in these genomes (Papke et al., 2004, 2007). These observations indicated gene flow as an important method for niche adaptation in these organisms. In Deep Lake, Antarctica the freezing temperatures limit the rate of replication to approximately 6 times per year and evolution in the halobacterial populations there mainly occurs through gene flow (Demaere et al., 2013). Recent whole genome comparisons revealed frequent gene transfer followed by homologous replacement of the transferred gene within the Halobacteria, hampering attempts to resolve the phylogeny within this group (Williams et al., 2012). Gene flow and recombination between populations and species make it difficult to resolve the species phylogeny among the different genera of Halobacteria (Papke et al., 2004). The use of gene concatenation in building reference trees, as exemplified by the ribosomal protein reference tree used in this work, has been pivotal in determining a branching order for the major clades of organisms, such as the Halobacteria, that participate in a large amount of recombination with close relatives. However, because genetic transfer and homologous recombination occur frequently between close relatives, the resulting phylogeny reflects both, shared ancestry and frequency of gene transfer. Therefore, determining the network of gene flow that overlays the vertical signal is important to the understanding of the evolution of these organisms. Inteins cannot penetrate the cell wall, and thus capitalize on existing gene flow in populations to efficiently invade when the opportunity presents itself. This trait can be exploited to keep track of successful homing events revealed by sequence similarity of inteins in distinct strains.

*Halorubrum* was the only genus in this study that had a large enough sample size to begin to uncover a signal reflecting population structure. Many of the *Halorubrum* genomes in this study were isolated from the same location, and this collection of genomes showed a clear signal for a structured population. Sixteen genomes from Aran-Bidgol were separated into four well-supported clusters. Three of the four clusters have branching orders identical to those in the reference tree, and the support values for those clusters could be attributed to both transfer within the group and a background phylogenetic signal or ancestral inheritance of similar intein alleles. However, only cluster 7 in the *Halorubrum* shares all intein alleles between all members of the cluster while the other clusters all contain intein alleles that are unique to certain members of the cluster, suggesting ongoing transfer of these inteins within the population. Additionally, three out of the twelve total clusters demonstrate unique branching orders compared to the reference tree, though only five of the clusters reflected in the reference tree have identical intein profiles. The lack of fixation for the intein alleles in the majority of clusters (seven out of twelve) indicates that a signal due to vertical inheritance may aid the formation of the clusters, but that HGT and its bias is the driving force for intein distribution. This analysis demonstrates the utility of intein sequences in distinguishing a population structure amongst genomes isolated from the same location, as demonstrated with the genomes isolated from Aran-Bidgol. These relationships are made evident through analyzing all of the signals from each of the intein alleles represented in the strains, and thus represent a collapsed view of the major gene sharing networks that have shaped the intein profiles of these strains over time. The collapsed networks indicate a higher rate of recombination within compared to between species and groups, a finding similar to the sexual outcrossing in fungal populations where inteins also thrive, as the semi-sexual lifestyle promotes intein homing (Giraldo-Perez and Goddard, 2013).

It is tempting to speculate that strains that harbor an abundance of intein alleles partake in more gene transfer than their counterparts without as many inteins; however, these two phenomena should not be expected to have a strict correlation as HGT between strains that possess only one intein each cannot produce hybrids with more than two inteins each. The number of inteins present in a group of different strains and species may be more reflective of transfers with divergent organisms than within-group transfer frequency.

The presented research demonstrates the utility of intein sequences to follow gene flow within and between populations. Improved reliability to assess the presence and activity of the HEN domain intein will provide a better distinction between vertical and horizontal inheritance of inteins. The overall utility of inteins improves as new intein alleles and new host proteins are reported, increasing the distribution of samples and improving statistical robustness of studies like the one done here. Prior to this work, nine proteins had been reported to contain inteins in the Halobacteria. This work established seven new intein alleles in the Halobacteria, including two proteins not previously reported to contain inteins. The presence of inteins is especially useful in populations where high rates of recombination and widely distributed populations may facilitate the maintenance of intein sequences over long periods of time (Gogarten and Hilario, 2006) and provide a means for distinguishing closely related partners involved in genetic transfers. The phylogenetic distribution of intein alleles, combined with the changing state within intein alleles, and the rapid substitution rate of inteins relative to the extein host sequences (Swithers et al., 2013) will provide a valuable tool to infer gene flow dynamics in and between sampled populations.

## Author contributions

Johann Peter Gogarten and Shannon M. Soucy participated in the design of this study and helped to draft the manuscript. Shannon M. Soucy performed the research and all authors contributed to data analysis. All authors read and approved the final manuscript.

### Conflict of interest statement

The authors declare that the research was conducted in the absence of any commercial or financial relationships that could be construed as a potential conflict of interest.
